# Atomic Pathways of Crystal-to-Crystal Transitions and Electronic Origins of Resistive Switching in MnTe for Ultralow-Power Memory

**DOI:** 10.3390/nano15030231

**Published:** 2025-01-31

**Authors:** Rui Wu, Nian-Ke Chen, Ming-Yu Ma, Bai-Qian Wang, Yu-Ting Huang, Bin Zhang, Xian-Bin Li

**Affiliations:** 1 State Key Laboratory of Integrated Optoelectronics, College of Electronic Science and Engineering, Jilin University, Changchun 130012, China; 2 Analytical and Testing Center, Chongqing University, Chongqing 401331, China

**Keywords:** phase change memory, MnTe, first-principles calculations, crystal-to-crystal transitions

## Abstract

In conventional phase change memory (PCM) technology, the melting process required to create an amorphous state typically results in extremely high power consumption. Recently, a new type of PCM device based on a melting-free crystal-to-crystal phase transition in MnTe has been developed, offering a potential solution to the problem. However, the electronic and atomic mechanisms underlying this transition remain unclear. In this work, by first-principles calculations, the resistance contrast is attributed to the differences in hole effective mass and vacancy formation energy of the two phases. Moreover, two phase transition pathways of the α-MnTe-to-β-MnTe transition, namely, the ‘slide-and-stand-up’ transitions, are identified based on coherent atomic movements. The energy barriers for the two pathways are 0.17 eV per formula unit (f.u.) and 0.38 eV/f.u., respectively. Furthermore, the energy barriers can be reduced to 0.10 eV/f.u. and 0.26 eV/f.u. via c-axis tensile strains, which makes the phase transition easier. The current result provides new insights into the non-melting phase transition process in MnTe, facilitating the development of low-power PCM technology.

## 1. Introduction

Phase change memory (PCM) has been regarded as a promising candidate for storage-class memory, embedded memory and computing-in-memory [1,2,3,4,5,6,7,8], owing to its outstanding performance with fast speed, good scalability and high reliability [9,10,11,12]. Unfortunately, the issue of high power consumption has long been a pain point for PCM, which limits its application in high-density integrated circuits [13,14]. The high power consumption of conventional PCM arises from the high-temperature melting of PCM materials during the crystalline-to-amorphous transition (RESET operation), which consumes too much energy [13,15]. Therefore, there has been a longstanding goal to achieve PCM through melting-free crystalline-to-crystalline phase transitions (*c-to-c* PCM) [16,17]. The idea has been proposed in various explorations such as the layer-block sequence transition in interfacial PCM [18], the electron beam-induced 2H-to-1T transition in MoS_2_ [19], the ultrafast laser-induced 2H-to-1T’ transition in MoTe_2_ [20,21], the ultrafast laser-induced rhombohedral-to-cubic transition of GeTe [22,23,24], and the thermally driven α-to-β and β-to-γ phase transitions in In_2_Se_3_ [25,26,27], among others. Despite these intriguing proposals and material-level demonstrations, *c-to-c* PCM devices with practical performance are still absent.

Recently, a new type of *c-to-c* PCM device utilizing the reversible phase transitions between α-MnTe and β-MnTe was realized by Sutou’s group [28]. The power consumption of the device was indeed an order of magnitude lower than that of conventional PCM devices. High-resolution TEM analyses also demonstrated that the transformation between α-MnTe and β-MnTe is a diffusionless displacement-type phase transition [29]. At present, the endurance of the MnTe-based *c-to-c* PCM device is limited to only a few hundred cycles. Therefore, optimizing the material composition, stimulation pulses and device architecture or exploring new materials with similar properties is urgently needed. The possible phase transition mechanism has been proposed in several reports by Mori et al. [28,29,30]. However, the resistive switching mechanism and the atomic pathways of the phase transitions between α-MnTe and β-MnTe are still not fully understood, primarily due to the lack of dynamic pictures of the transition processes at atomic scales, which hinders the optimization and design of materials for *c-to-c* PCM devices. Theoretical investigations on the experimental observations should be helpful to understand the underlying mechanisms.

In this work, we uncover the electronic origins of resistive switching, the atomic pathways of the *c-to-c* transitions and the effect of strain on these transitions through first-principles calculations. The resistance contrast between the two phases is attributed to differences in hole effective mass and vacancy defect formation energy. Atomic structure analyses have clarified that the previously hypothesized intermediate phase is possibly unlikely to exist. Two ‘*slide-and-stand-up*’ pathways of the transitions from α-MnTe to β-MnTe are identified through transition state analyses, with energy barriers of approximately 0.17 eV per formula unit (eV/f.u.) and 0.38 eV/f.u. for *Path 1* and *Path 2*, respectively. Moreover, the energy barriers can be modulated by stress or strain. By applying experimentally feasible uniaxial strains along the c-axis, the energy barriers of the α-MnTe-to-β-MnTe transition can be reduced to 0.10 eV/f.u. and 0.26 eV/f.u. for *Path 1* and *Path 2*, respectively. In contrast, the β-MnTe-to-α-MnTe transitions are only slightly affected by the strains. The revealed electronic and atomic mechanisms not only explain the performance of MnTe-based *c-to-c* PCM devices but also offer guidance for optimizing low-energy-cost PCM materials and device designs.

## 2. Computational Methods

**Figure 1:**
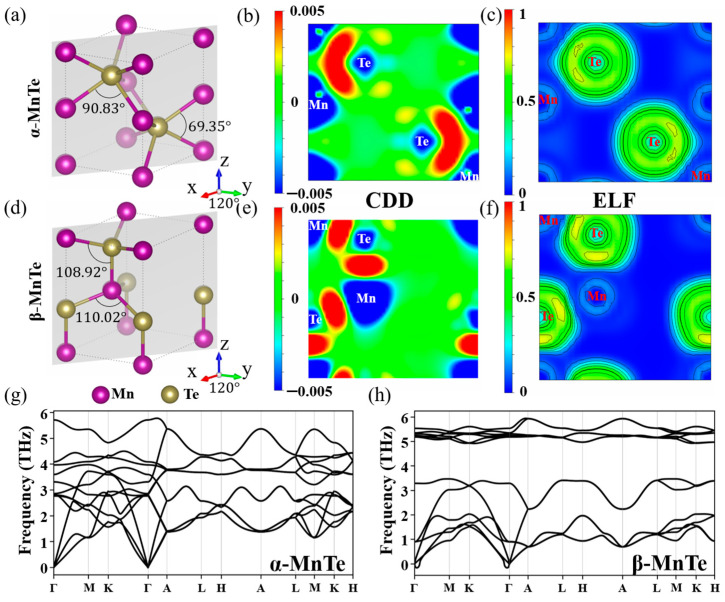
Atomic structure, charge density difference (CDD) and electron localization function (ELF) of (**a**–**c**) α-MnTe and (**d**–**f**) β-MnTe. The (110) cross-section is selected to show the CDD and ELF. The unit of CDD is *e*/*a*_0_^3^; *a*_0_ is Bohr radius. (**g**,**h**) Phonon spectra of α- and β-MnTe, respectively.

The first-principles calculations in this work are performed using VASP code based on density functional theory (DFT) [31]. The Perdew–Burke–Ernzerhof (PBE) functional with generalized gradient approximation (GGA) is used to evaluate the exchange correlation effects [32]. Non-local corrections are applied by the GGA + U approach with U = 3 eV [33]. The plane wave cutoff energy is 350 eV. The k-points for structure relaxation and electronic structure calculations are 9 × 9 × 9 and 11 × 11 × 11, respectively. The residual force convergence criterion for structure relaxation is 0.01 eV·Å^−1^. The energy is considered to have converged as two consecutive steps with an energy variation of less than 10^−6^ eV. To set the anti-ferromagnetic orders, the unit cell of our MnTe model contains two Mn atoms and two Te atoms. The mass densities of the α-MnTe and β-MnTe models are 5.94 g/cm^3^ and 4.54 g/cm^3^, respectively. The lattice directions of the crystal are indicated by the crystallographic axes in Figure 1. The phonon spectra are calculated using the DFPT method as implemented in the Phonopy code [34]. The effective masses are analyzed using the EMC code [35]. The energy barriers are calculated using the Climbing Image Nudged Elastic Band (CI-NEB) method [36]. The lattice constants are fixed during the NEB analyses. The band structures and density of states (DOSs) are drawn by the Pymatgen code [37]. The structures and charge densities are visualized by the VESTA code [38]. 

## 3. Results and Discussions

According to previous experiments, the two phases employed as the low-resistance state (LRS) and high-resistance state (HRS) in the *c-to-c* PCM device are the NiAs-type hexagonal α phase and the wurtzite-type β or strained-β (β’) phase of MnTe, respectively [28]. The atomic structures of α-MnTe and β-MnTe are shown in Figure 1a,d. Both phases exhibit antiferromagnetic order and are therefore treated as A-type collinear antiferromagnetic materials in the following calculations [39,40]. The magnetic moments at the Mn sites for the two phases are shown in Table S1 of the Supplementary Materials. The calculated lattice parameters (a = b = 4.20 Å, and c = 6.68 Å for α-MnTe and a = b = 4.54 Å, and c = 7.36 Å for β-MnTe) agree well with the experimental results [28]. The analyses on the charge density difference (CDD) and electron localization function (ELF) in Figure 1b,c,e,f indicate that the bonding properties of α-MnTe and β-MnTe are similar. The accumulation of electrons between Mn and Te atoms indicates a covalent bonding component, while the electron transfer from Mn to Te suggests an ionic bonding component. The calculated phonon spectra in Figure 1g,h demonstrates that both α-MnTe and β-MnTe are stable without imaginary frequencies. Also, the optical modes of β-MnTe are more localized than those of α-MnTe, which suggests the bonds of β-MnTe are more rigid.

**Figure 2:**
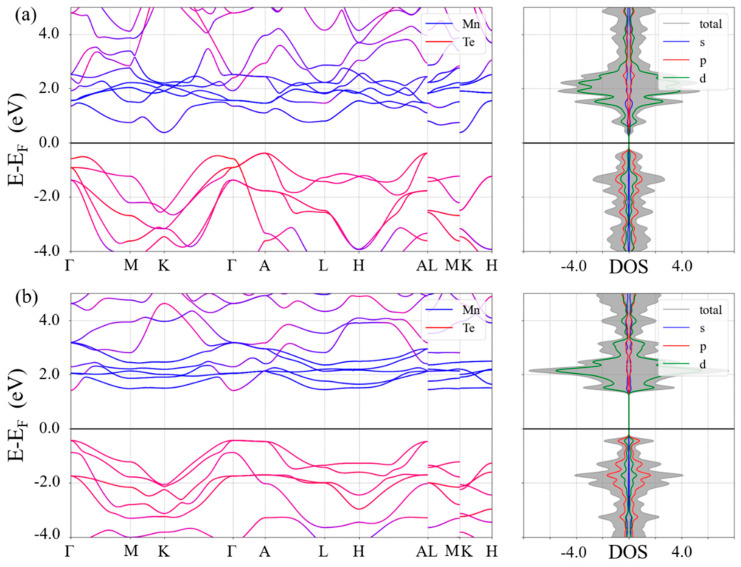
Element resolved band structures (left panels) and density of states (right panels) of (**a**) α-MnTe and (**b**) β-MnTe. The color coding refers to the contribution of the Mn (blue) and Te (red) elements. Mixed contributions are represented by intermediate colors.

**Figure 3:**
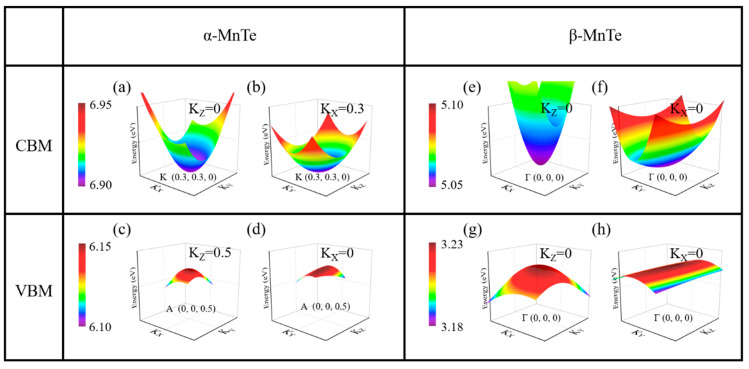
Three-dimensional *E-k* diagrams near CBM and VBM of (**a**–**d**) α-MnTe and (**e**–**h**) β-MnTe.

The experimentally measured band gaps of α-MnTe (about 1.25–1.51 eV) and β-MnTe (about 2.7 eV) are different [41,42,43]. However, the resistances of the two phases are not directly determined by the band gaps, as the carrier concentrations are significantly higher than those arising from intrinsic excitation [28]. To elucidate the origin of the resistance contrast between α-MnTe and β-MnTe, the analysis of their electronic structures is required. Figure 2 presents the calculated band structures and density of states (DOSs) for the two phases, revealing that α-MnTe exhibits an indirect band gap of 0.76 eV, while β-MnTe features a direct band gap of 1.84 eV. These results are consistent with the experimental findings, considering the general underestimation of the band gap by DFT. In addition, we also calculated the band structure, the density of states and the total energy using larger values of U (Figure S1 and Table S2 in the Supplementary Materials). The band structures and energy differences between α-MnTe and β-MnTe are only slightly affected. Moreover, we further performed calculations using the hybrid functional (HSE06), which better reproduces the experimental band gaps, revealing that α-MnTe exhibits an indirect band gap of 1.43 eV, while β-MnTe features a direct band gap of 2.58 eV (Figure S2 in the Supplementary Materials). The spin–orbit coupling effect is also demonstrated to have tiny influences on the band structures (see Figure S3 in the Supplementary Materials). The DOS indicates that the states near the conduction band minimum (CBM) are mainly contributed by *3d* orbitals of Mn atoms, while those near the valence band maximum (VBM) are mainly composed of *p* orbitals of Te atoms. According to the band structures, the band near the VBM of β-MnTe appears flatter than that of α-MnTe, suggesting a larger effective mass and potentially lower carrier mobility. To verify this, three-dimensional *E*-*k* diagrams near the CBM and VBM of the two phases were calculated (Figure 3). Indeed, the 3D *E-k* relations show a very flat band in β-MnTe (Figure 3h). To provide a quantitative assessment, the effective masses of electrons at the CBM and holes at the VBM were calculated using the EMC code [35]. Table 1 shows the calculated results. The average electron effective mass (*m*_n_*) at CBM of α-MnTe is larger than that of β-MnTe, while the average hole effective mass at VBM (*m*_p_* = −*m*_n_*) of α-MnTe is smaller than that of β-MnTe. More details of the directions of the effective masses are presented in Table S3 of the Supplementary Materials. Since both phases exhibit *p*-type conductivity, the smaller hole effective mass of α-MnTe leads to higher carrier mobility compared to β-MnTe, consistent with experimental observations [41,44].

As for the carrier concentration, it has been reported that the *p*-type conductivity of MnTe comes from the Mn vacancy (V_Mn_) [45], suggesting that the concentration of V_Mn_ defects governs the carrier concentrations. Then, we calculate the formation energies of V_Mn_ in α-MnTe and β-MnTe (see Note S1 and Figure S4 in the Supplementary Materials for more details). The results indicate that the formation energies of V_Mn_ in α-MnTe (0.36–3.25 eV) are indeed lower than those in β-MnTe (1.58–4.49 eV). Based on the formation energy of V_Mn_, we estimate the ratio of V_Mn_ concentration in β-MnTe to that in α-MnTe (Note S1 and Figure S5 in the Supplementary Materials) [46]. The V_Mn_ concentration in β-MnTe can be several orders of magnitude smaller than that in α-MnTe, suggesting that the hole concentration will be significantly reduced after the α-to-β transitions. Therefore, we propose that the concentration of V_Mn_ decreases after the α-to-β transition in MnTe, which in turn reduces the carrier concentrations. In brief, the origin of the resistive switching after the α-to-β transition in MnTe is attributed to the increased hole effective mass and the enhanced formation energy of V_Mn_. 

**Figure 4:**
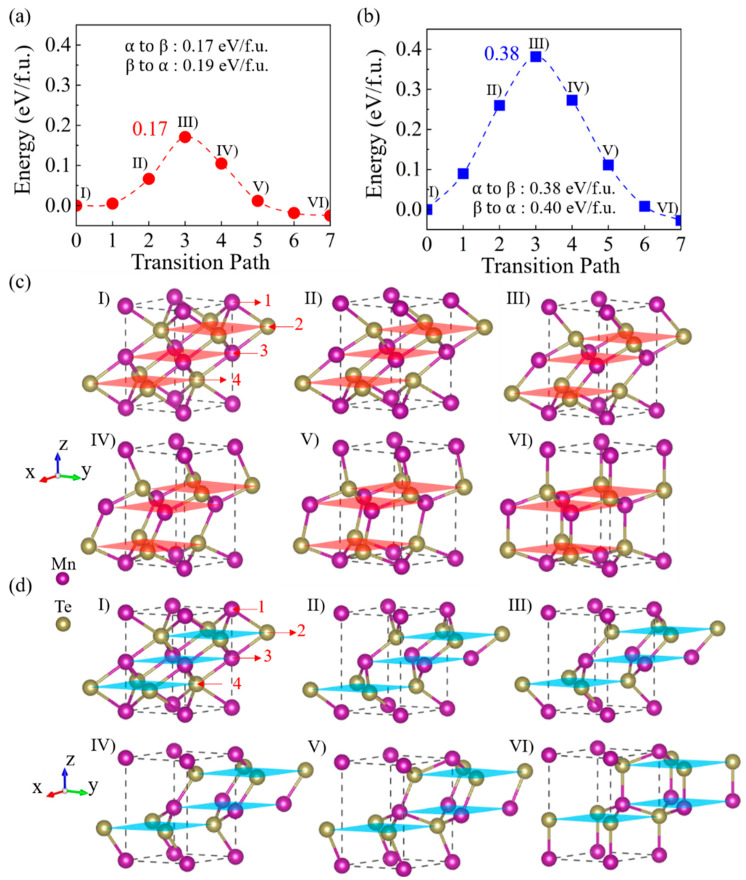
The energy landscapes during the phase transitions from α-MnTe to β-MnTe via (**a**) *Path 1* and (**b**) *Path 2*. Snapshots of the structures corresponding to states I–VI in (**c**) *Path 1* and (**d**) *Path 2*. The shaded slices indicate the crystal planes of different atomic layers. The arrows indicate the moving directions of the atomic layers. Among them, the purple symbols correspond to Mn atoms, and the yellow symbols correspond to Te atoms.

Understanding the atomic pathway of phase transitions between α-MnTe and β-MnTe is essential for comprehending and controlling the *c-to-c* transitions. Here, the CI-NEB method is used to identify the transition states of the α-to-β phase transition, by which two possible pathways are found (Figure 4). Figure 4a illustrates the energy landscape of *Path 1*, with an energy barrier of approximately 0.17 eV/f.u. Six atomic configurations along the transition path (labeled I to VI) are shown in Figure 4c. These consecutive atomic snapshots reveal that the process is a displacement-type transition without long-range atomic diffusions. During the transition, the second-layer (Te) and third-layer (Mn) atoms slide along the $[1\bar{1}0]\;$ direction (from right to left in Figure 4c), while the first-layer (Mn) and fourth-layer (Te) atoms move in the opposite direction (from left to right in Figure 4c). To complete this process, two Te-Mn bonds around each atom are broken. Then, the six-coordinated configuration in α-MnTe turns into the four-coordinated tetrahedral configuration in β-MnTe. The process seems like a ‘*slide-and-stand-up*’ motion of the Te-Mn bonds between the first-layer (Mn) and second-layer (Te) atoms, as well as between the third-layer (Mn) and fourth-layer (Te) atoms. As for the transition from β-MnTe to α-MnTe, it should occur in the reverse manner with an energy barrier of 0.19 eV/f.u. (Figure 4a).

Note that the ‘*slide-and-stand-up*’ pathway of *Path 1* involves the sliding of atoms along the [1$\bar{1}$0] and [$\bar{1}$10] directions, resembling the two-step process (α-β′-β) proposed in previous experimental studies [29]. However, the ‘*slide-and-stand-up*’ motion is directly accomplished through the sliding and rotation of chemical bonds, rather than the previously proposed two-step ‘*slide-and-expansion*’ or ‘*buckling-and-puckering*’ process [28,29]. Then, further calculations are performed to check the stability of the proposed intermediate β′ phase. Figure S6a shows the structure of the β′ phase, adopting the lattice constant of the α phase as depicted in a previous report [28]. It immediately turns into the β phase after structural relaxations (Figure S6b). Figure S6c shows the atomic forces on different atoms. The large atomic forces suggest that the structure cannot be stable. The situation is the same when the β′ phase adopts the lattice constant of the β phase (Figure S6d). This instability is physically reasonable, as some chemical bonds in the proposed β′ phase are significantly compressed, leading to strong Coulomb repulsions that hinder the stabilization. Therefore, further experimental investigations are needed in future studies to address the issue.

We notice that the α-to-β phase transition can also be realized by the sliding motions in the opposite directions to those of *Path 1*. *Path 2* is then constructed by displacing the atoms in the opposite directions to those in *Path 1*. The transition states of *Path 2* are also identified by the NEB method. Figure 4b shows the energy landscape of α-to-β transition via *Path 2*, with an energy barrier of 0.38 eV/f.u. The first half of the pathway is shown in Figure 4d (states I–III), where the second-layer (Te) and third-layer (Mn) atoms move along the [$\bar{1}$10] direction, while the first-layer (Mn) and fourth-layer (Te) atoms move in opposite directions. For the second half of the pathway (states IV–VI in Figure 4d), the third-layer (Te) and fourth-layer (Mn) atoms move along the [$\bar{1}$10] direction, leading to the rotation of Te-Mn bonds between the second-layer (Te) and third-layer (Mn) atoms, as well as between the fourth-layer (Te) and fifth-layer (Mn) atoms. During the transition, up to three chemical bonds around each atom are broken (see Figure 4d, state III). Therefore, the energy barrier is larger than that of *Path 1*. Note that a β′-like transition state is observed in *Path 2*, as illustrated in Figure S6e (i.e., state III in Figure 4d). The forces on the atoms are negligible in this structure (Figure S6f) because the structure corresponds to the saddle point of the energy landscape (i.e., state III in Figure 4b). No chemical bonds are compressed in such a β′-like structure. Therefore, state III and the nearby states, such as state II in Figure 4d, may be stabilized by constraints from the surrounding matrix. Further experimental explorations are needed to clarify whether these β′-like structures are related to the previously observed β′ phase.

Next, we study the effect of stress on the *c-to-c* transition in MnTe because considerable stresses may exist due to device constraints or thermal expansion effects. For instance, it has been reported that the c-axis lattice constant of the β′ phase is very close to that of α phase, indicating that the β′-MnTe is a strained phase under compression stress [28]. Similarly, another report shows that the c-axis lattice constant of the α phase near the α-MnTe/β-MnTe interface after the β-to-α transition is close to that of β-MnTe, suggesting it is a strained phase under tensile stress [47]. Therefore, the effects of stress and strain on the phase transition warrant further investigation.

**Figure 5:**
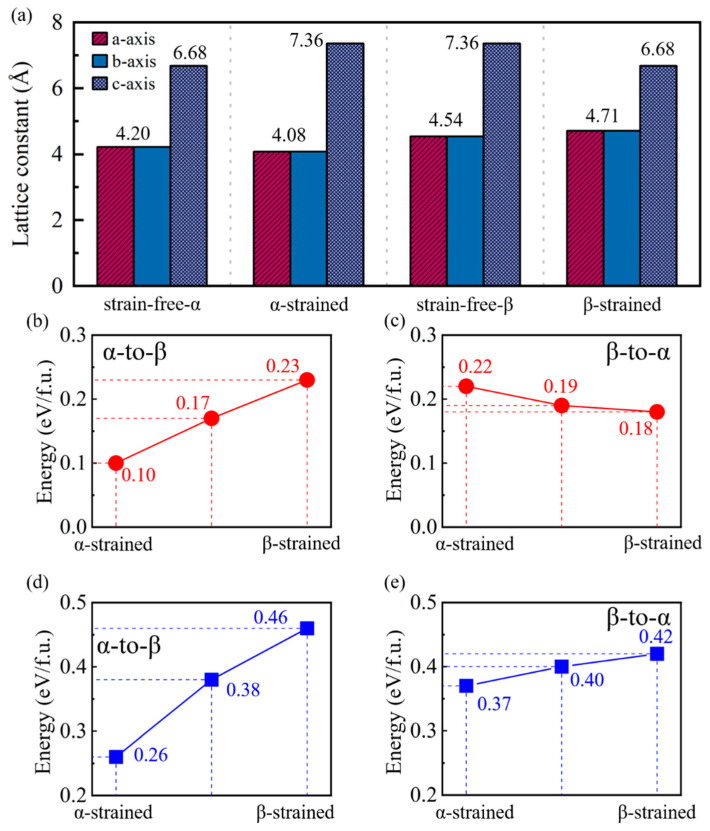
(**a**) The lattice constants of the strained and strain-free α-MnTe and β-MnTe that used as the initial and final states in the NEB calculations. The calculated energy barriers of the α-to-β and β-to-α phase transitions via (**b**,**c**) *Path 1* and (**d**,**e**) *Path 2* under different strain conditions.

According to experimental reports [28,47], two strain or stress conditions are considered (Figure 5a): (1) the α-strained condition, where the c-axis lattice constant of α-MnTe is strained to match that of β-MnTe during the transition, and (2) the β-strained condition, where the c-axis lattice constant of β-MnTe is strained to match that of α-MnTe during the transition. Note that under both strained conditions, the lattice constants of α-MnTe and β-MnTe along the a- and b-axes are slightly relaxed due to Poisson’s effect (see Figure 5a). CI-NEB calculations are then performed for the transitions under the two strained conditions: from α-strained α-MnTe to strain-free β-MnTe and from strain-free α-MnTe to β-strained β-MnTe (see Figures S7 and S8). The energy barriers of the phase transitions are altered, while the transition pathways remain unchanged (Figure 5b–e). For *Path 1* under the α-strained condition (Figure 5b), the energy barrier for the α-to-β transition decreases significantly to 0.10 eV/f.u., while the barrier for the β-to-α transition increases slightly to 0.22 eV/f.u. (Figure 5c). In contrast, under the β-strained condition, the energy barrier for the α-to-β transition via *Path 1* increases to 0.23 eV/f.u. (Figure 5b), while the barrier for β-to-α transition decreases slightly to 0.18 eV/f.u. (Figure 5c). For *Path 2*, the α-strained condition not only significantly reduces the energy barrier for α-to-β transitions to 0.26 eV/f.u. (Figure 5d) but also slightly reduces the barrier for β-to-α transition to 0.37 eV/f.u (Figure 5e). The β-strained condition in turn enhances both of the barriers for α-to-β and β-to-α transitions. According to ref. [28], the temperature for α-to-β transition is higher than that for β-to-α transition. Therefore, we suggest introducing tensile strains along the c-axis in the device to further reduce the RESET voltage. The lattice of β-MnTe under the z-direction compression condition is relaxed along the x-y directions. If the lattice is not relaxed along the x-y directions, the total energy of the β-MnTe will be 0.18 eV/f.u. larger than the energy of α-MnTe. The energy difference between α-MnTe and β-MnTe is smaller than that in a recent experiment [30]. The reason may be that the calculations are performed at 0K. Further investigations at finite temperatures are needed to clarify the issue. 

## 4. Conclusions

In summary, first-principles calculations were employed to investigate the electronic origin of resistive switching and the phase transition pathways between α-MnTe and β-MnTe. The hole effective mass of β-MnTe (HRS) is larger than that of α-MnTe (LRS), resulting in lower carrier mobility. Additionally, the formation energy of V_Mn_ defects in β-MnTe is higher than that in α-MnTe, leading to a reduced hole concentration. Consequently, β-MnTe exhibits a higher resistance than α-MnTe. Using the NEB method, two ‘*slide-and-stand-up*’ transition pathways from α-to-β with energy barriers of 0.17 eV/f.u. and 0.38 eV/f.u. were identified. These transitions occur through coherent sliding of atomic layers accompanied by the breaking and rotation of chemical bonds. Furthermore, tensile strain along the c-axis is shown to significantly reduce the energy barriers for the α-to-β transitions, while having only a slight effect on the β-to-α transitions. These findings provide a detailed electronic and atomic understanding of the α-to-β transition in MnTe, offering valuable insights for optimizing the design of *c-to-c* PCM materials and devices with low power consumption.

## Figures and Tables

**Table 1:** Electron effective masses in different directions at the CBM and VBM of α-MnTe and β-MnTe.

	α-CBM	α-VBM	β-CBM	β-VBM
$m_{1}^{*}$	0.352	−0.326	0.209	−0.915
$m_{2}^{*}$	0.352	−0.459	0.058	−0.918
$m_{3}^{*}$	0.259	−3.120	0.042	−7.550

## Data Availability

The original contributions presented in the study are included in the article and Supplementary Materials. Further inquiries can be directed to the corresponding author.

## Supplementary Materials

The following supporting information can be downloaded at https://www.mdpi.com/article/10.3390/nano15030231/s1, Table S1. The magnetic moments at the Mn sites for α-MnTe and β-MnTe. The unit is $\mu_{B}$. Figure S1. (a) The band structures and density of states of α-MnTe and β-MnTe calculated using the GGA + U (U = 4 eV) method. (b) The band structures and density of states of α-MnTe and β-MnTe calculated using the GGA + U (U = 5 eV) method. Table S2. The band gap and total energy of MnTe calculated using different values of U. Figure S2. The band structures of α-MnTe and β-MnTe calculated using the HSE06 method. Figure S3. Band structures of α-MnTe and β-MnTe calculated with the spin–orbit coupling (SOC) effect. Table S3. The corresponding eigenvectors of the principal effective masses. Note S1. Calculation details of formation energy and concentration of V_Mn_. Figure S4. Neutral formation energy of Mn vacancy in α-MnTe and β-MnTe as a function of chemical potential μ_Mn_. Figure S5. The ratio of the V_Mn_ concentrations in β-MnTe to that in α-MnTe as a function of temperature at their equilibrium states. Figure S6. (a) The structure of the β′ phase as depicted in the previous reports. The β′ phase is defined as the vertical distance between Mn and Te atoms along the c-axis in the ratio of 1:1. However, after structural relaxation, the ratio becomes 3:1, which corresponds to (b) the standard β phase. The forces on Mn and Te atoms in β′ phases with different lattice constants: (c) the c-axis lattice constant of β′ phase adopts that of the α phase; (d) the c-axis lattice constant of the β′ phase adopts that of the β phase. The extremely large atomic forces suggest the β′ phase is not stable. (e) The structure of the β′-like transition state in Path 2 (i.e., state III of Figure 4d in the main text). (f) The forces on atoms in the β′-like phase. Figure S7. The energy landscapes of the phase transitions via Path 1 under the two strained conditions: (a) from α-strained α-MnTe to strain-free β-MnTe (i.e., α-strained condition) and (b) from strain-free α-MnTe to β-strained β-MnTe (i.e., β-strained condition). Figure S8. The energy landscapes of the phase transitions via Path 2 under the two strained conditions: (a) from α-strained α-MnTe to strain-free β-MnTe (i.e., α-strained condition) and (b) from strain-free α-MnTe to β-strained β-MnTe (i.e., β-strained condition).
