# Supplementary material for: Atomic Pathways of Crystal-to-Crystal Transitions and Electronic Origins of Resistive Switching in MnTe for Ultralow-Power Memory
Source: Nanomaterials (Basel). 2025 Jan 31;15(3):231. doi: 10.3390/nano15030231 (PMC11820569; doi:10.3390/nano15030231)
Supplement: Supplementary file 1 [file nanomaterials-15-00231-s001.zip › nanomaterials-3410802-supplementary.pdf]

Supplementary Material

Atomic Pathways of Crystal-to-Crystal Transitions and Electronic Origins of Resistive Switching in MnTe for Ultralow-Power Memory

Rui Wu[1], Nian-Ke Chen[1,*], Ming-Yu Ma[1], Bai-Qian Wang[1], Yu-Ting Huang[1], Bin Zhang[2], and Xian-Bin Li[1,*]

[1]*State Key Laboratory of Integrated Optoelectronics, College of Electronic Science and Engineering, Jilin University, Changchun 130012, China*

[2]*Analytical and Testing Center, Chongqing University, Chongqing 401331, China*

**Author to whom any correspondence should be addressed.*

*Email: chennianke@jlu.edu.cn or lixianbin@jlu.edu.cn

Table S1. The magnetic moments at the Mn sites for α-MnTe and β-MnTe. The unit is μ_B.

α-MnTe		β-MnTe	
Mn1	4.438	Mn1	4.505
Mn2	-4.421	Mn2	-4.505

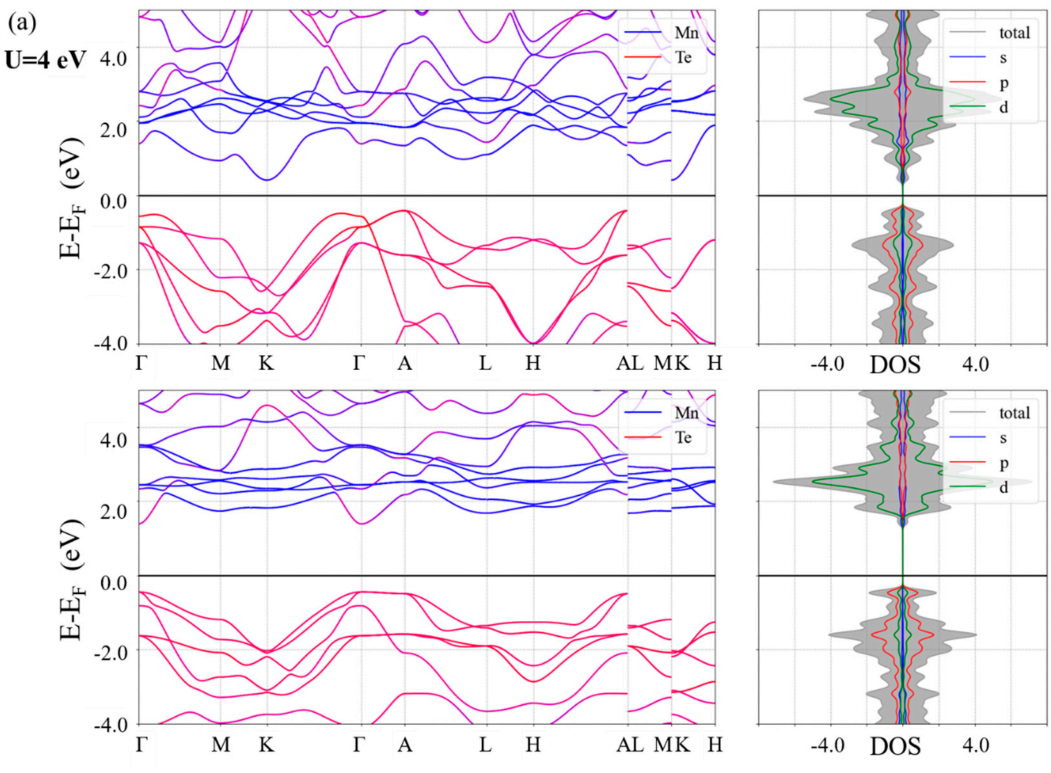

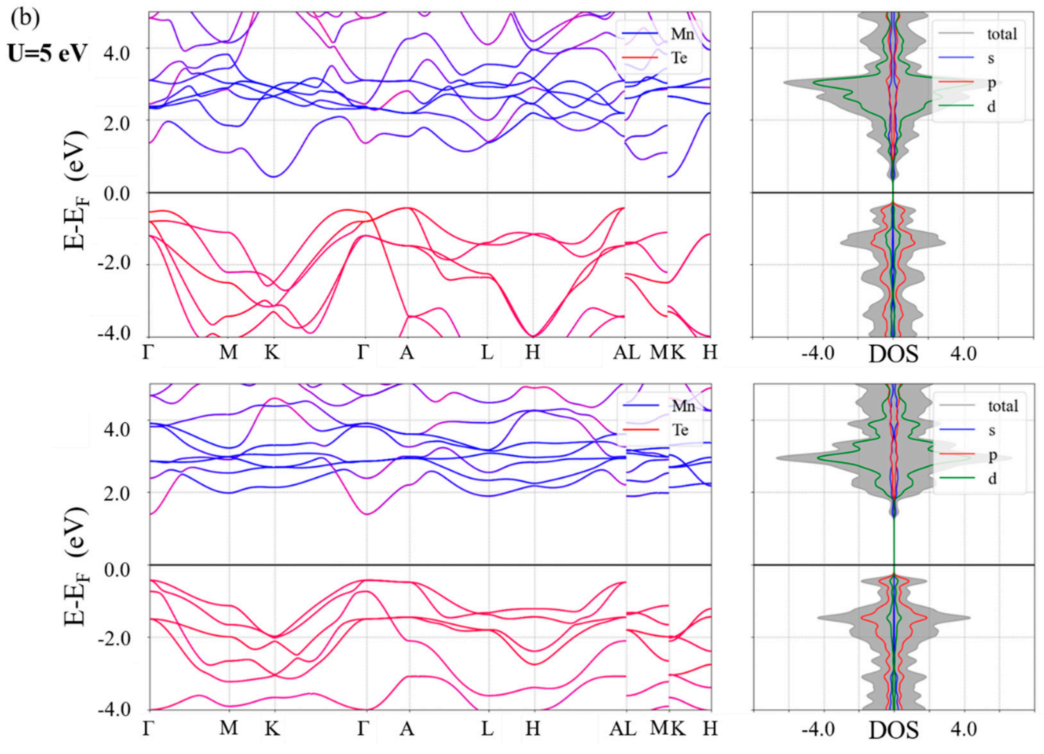

Figure S1. (a)The band structures and density of states of α-MnTe and β-MnTe calculated using GGA+U (U = 4 eV) method. (b)The band structures and density of states of α-MnTe and β-MnTe calculated using GGA+U (U = 5 eV) method.

Table S2. The bandgap and total energy of MnTe calculated using different values of U.

	α-bandgap (eV)	β-bandgap (eV)	Eα (eV)	Eβ (eV)
U = 3 eV	0.76	1.84	-22.72	-22.76
U = 4 eV	0.82	1.83	-22.18	-22.26
U = 5 eV	0.85	1.82	-21.71	-21.80

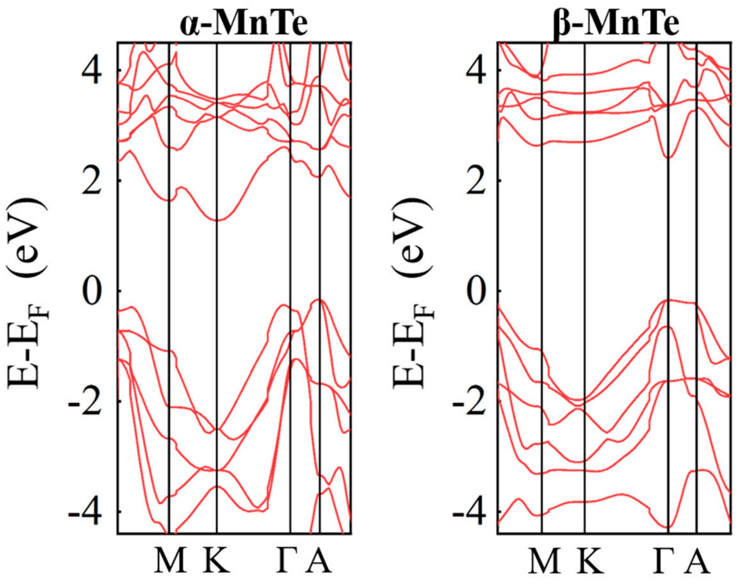

Figure S2. The band structures of α-MnTe and β-MnTe calculated using the HSE06 method.

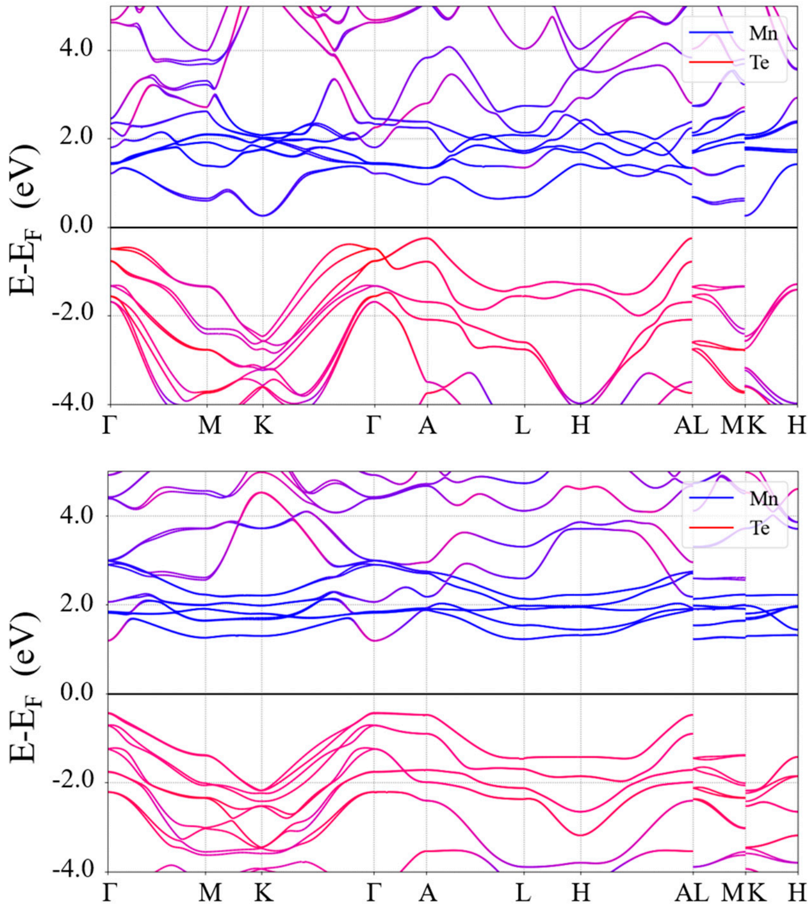

Figure S3. Band structures of α-MnTe and β-MnTe calculated with the spin-orbit coupling (SOC) effect.

The effective mass tensor can be expressed as

$$\frac{d^2E}{dk^2} = \begin{pmatrix} \frac{d^2E}{dk_x^2} & \frac{d^2E}{dk_x dk_y} & \frac{d^2E}{dk_x dk_z} \\ \frac{d^2E}{dk_x dk_y} & \frac{d^2E}{dk_y^2} & \frac{d^2E}{dk_y dk_z} \\ \frac{d^2E}{dk_x dk_z} & \frac{d^2E}{dk_y dk_z} & \frac{d^2E}{dk_z^2} \end{pmatrix},$$

where x, y, z are the directions in the reciprocal Cartesian space. The angle between the k_x direction and the k_y direction is 60 degrees for a hexagonal lattice. Eigenvalues of the above matrix are inverses of the effective masses, eigenvectors are the directions of the principal effective mass components (m_1^*, m_2^*, m_3^*). The eigenvectors of the principal effective masses (keeping one significant figure after the decimal point) are presented in Table S3.

Table S3. The corresponding eigenvectors of the principal effective masses.

	α-CBM	α-VBM	β-CBM	β-VBM
m_1^*	0.352	-0.326	0.209	-0.915
Eigenvectors for m_1^*	[-0.9, 0.4, 0.0]	[0.0, 0.8, 0.6]	[-0.6, 0.4, -0.7]	[0.1, 1.0, 0.1]
m_2^*	0.352	-0.459	0.058	-0.918
Eigenvectors for m_2^*	[0.4, 0.9, 0.0]	[1.0, 0.0, 0.0]	[0.4, 0.9, 0.2]	[1.0, -0.1, 0.0]
m_3^*	0.259	-3.120	0.042	-7.550
Eigenvectors for m_3^*	[0.0, 0.0, 1.0]	[0.0, -0.6, 0.8]	[-0.7, 0.2, 0.7]	[0.0, 0.1, 1.0]

Note S1. Calculation details of formation energy and concentration of V_{Mn}

For both α-MnTe and β-MnTe, the supercells of 4×4×3 are used to include the defects of Mn vacancies. The neutral formation energy for a defect d is defined as

$$\Delta H_f\,(d) = E(d) - E(host) + \sum_i n_i(E_i + \mu_i)$$

where $E(d)$ is the total energy of the supercell containing a defect d, and $E(host)$ is the total energy of the perfect host supercell. n_i is the number of atoms of element i being exchanged during the defect's formation between the host supercell and the atom reservoir with the energy $E_i + \mu_i$, where E_i is the energy per atom in the stable phase

of element i, and μ_i is the chemical potential with respect to E_i.

The chemical potentials of Mn (μ_{Mn}) and Te (μ_{Te}) atoms in MnTe are correlated by the enthalpy of formation of MnTe compounds $\Delta H(MnTe)$ with respect to the elementary substances, which is defined as

$$\Delta H\ (MnTe)\ =\ E(MnTe) -\ [E(Mn) + E(Te)],$$

where $E(MnTe)$ is the energy per formula unit of MnTe cell, $E(Mn)$ and $E(Te)$ are the energy per atom of Mn and Te elementary substances, respectively. The calculated $E(Mn)$ and $E(Te)$ are -5.33 eV/atom and -3.14 eV/atom, respectively. The calculated enthalpy of formation of α-MnTe and β-MnTe are -2.89 eV/f.u. and -2.91 eV/f.u., respectively.

Then, the chemical potentials of Mn and Te should satisfy the relationships of

$$\Delta H\ (MnTe) = \mu_{Mn} + \mu_{Te},$$

$$\mu_{Mn} \leq 0,$$

$$\mu_{Te} \leq 0.$$

The upper limits of the chemical potentials are $\mu_{Te} = 0$ or $\mu_{Mn} = 0$, which correspond to the Mn-poor (Te-rich) or Mn-rich (Te-poor) conditions, respectively. Then we calculated the chemical potential-dependent formation energy of a V_{Mn} defect (n_i=1).

For α-MnTe, the formation energy of V_{Mn}, $\Delta H_f\ (V_{Mn}\_\alpha)$ is calculated by

$$\Delta H_f\ (V_{Mn}\_\alpha) = E(V_{Mn}\_\alpha) -\ E(host\_\alpha) + (E_{Mn} + \mu_{Mn}),$$

$$\Delta H\ (\alpha MnTe) \leq \mu_{Mn} \leq 0.$$

Then we get

$$\Delta H_f\ (V_{Mn}\_\alpha) = 3.25 + \mu_{Mn},$$

$$-2.89 \leq \mu_{Mn} \leq 0.$$

For β-MnTe, the formation energy of V_{Mn}, $\Delta H_f\ (V_{Mn}\_\beta)$ is calculated by

$$\Delta H_f\ (V_{Mn}\_\beta) = E(V_{Mn}\_\beta) - \ E(host\_\beta) + (E_{Mn} + \mu_{Mn}),$$

$$\Delta H\ (\beta MnTe) \leq \mu_{Mn} \leq 0.$$

Then we get

$$\Delta H_f\ (V_{Mn}\_\beta) = 4.49 + \mu_{Mn},$$

$$-2.91 \leq \mu_{Mn} \leq 0.$$

The results of the chemical potential-dependent formation energy of V_{Mn} are shown in Figure S4. The formation energies of V_{Mn} in α-MnTe are always larger than those in β-MnTe under the same conditions.

The number of vacancies (*n*) at equilibrium states can be estimated by the following equation s[1]:

$$n = N \cdot \exp\left(-\frac{E_f}{k_B T}\right)$$

where N is the number of lattice sites of Mn, E_f is the formation energy of V_{Mn}, k_B is the Boltzmann constant, T is the temperature. The formation energy of V_{Mn} in β-MnTe (E_{f_β}) is 1.24 eV lager than that (E_{f_α}) in α-MnTe (Figure S4). Then the ratio of the V_{Mn} concentrations in β-MnTe and α-MnTe can be expressed as

$$\frac{n_\beta}{n_\alpha} = \frac{N \cdot \exp\left(-\frac{E_{f\_\beta}}{k_B T}\right)}{N \cdot \exp\left(-\frac{E_{f\_\alpha}}{k_B T}\right)} = \exp\left(-\frac{E_{f\beta} - E_{f\alpha}}{k_B T}\right)$$

Then the calculated results are shown in Figure S5. At equilibrium states, the V_{Mn} concentration in β-MnTe is much smaller than that in α-MnTe.

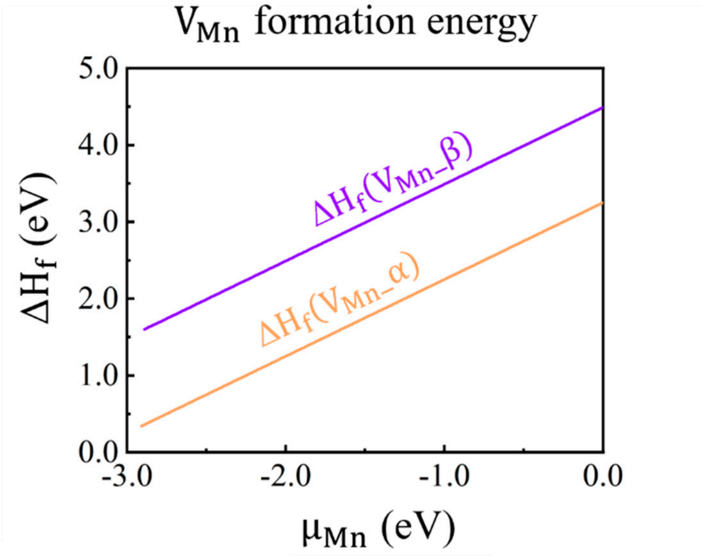

Figure S4. Neutral formation energy of Mn vacancy in α-MnTe and β-MnTe as a function of chemical potential μ_{Mn}.

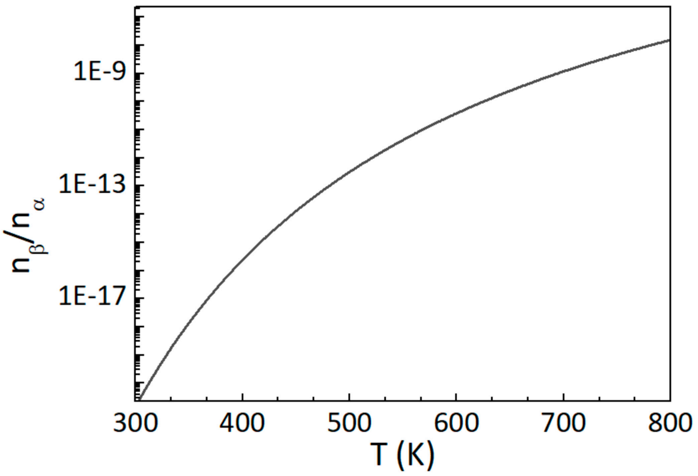

Figure S5. The ratio of the V_{Mn} concentrations in β-MnTe to that in α-MnTe as a function of temperature at their equilibrium states.

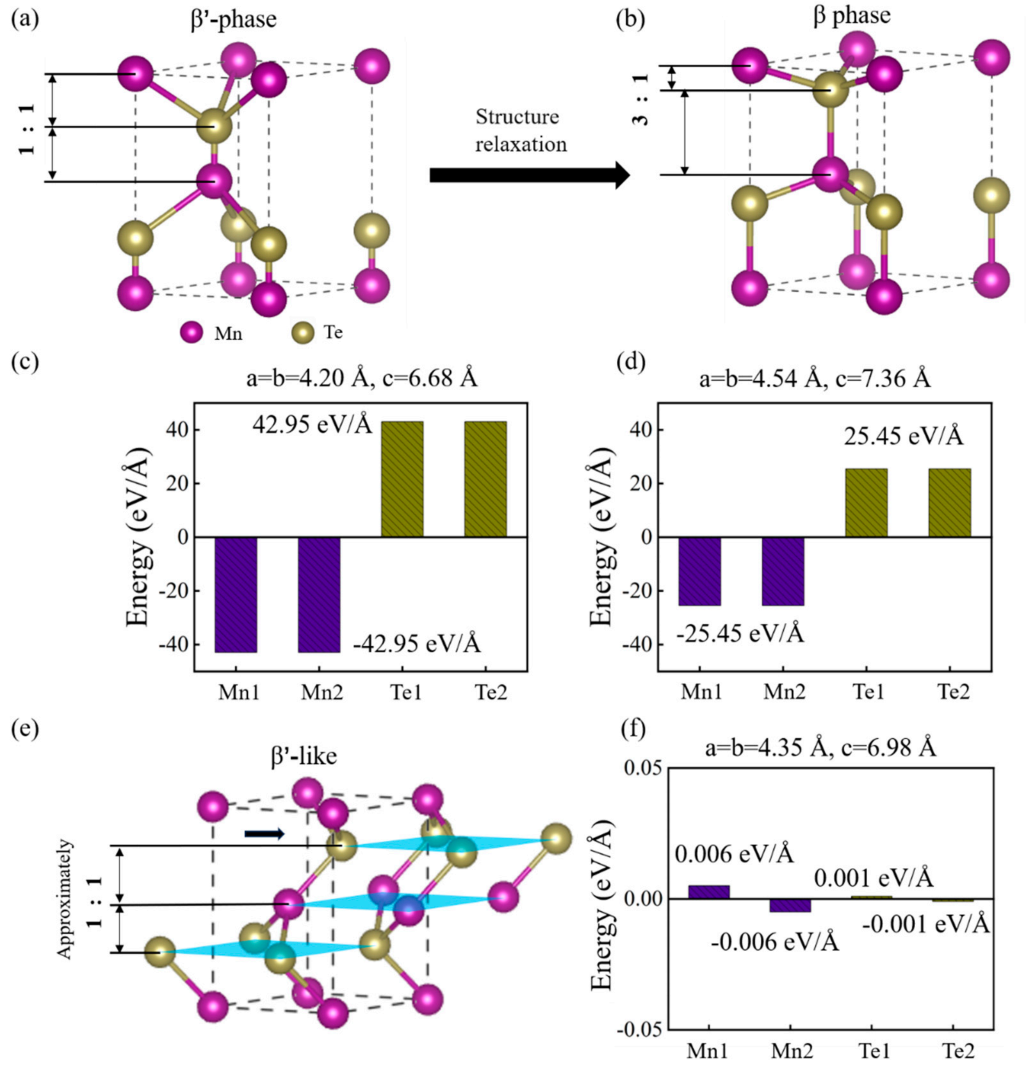

Figure S6. (a) The structure of the β′ phase as depicted in the previous reports.[s][2,3] The β′ phase is defined as that the vertical distance between Mn and Te atoms along the c-axis is in the ratio of 1:1. However, after structural relaxation, the ratio becomes 3:1, which corresponds to (b) the standard β phase. The forces on Mn and Te atoms in β′ phases with different lattice constants: (c) the c-axis lattice constant of β′ phase adopts that of α phase, (d) the c-axis lattice constant of β′ phase adopts that of β phase. The extremely large atomic forces suggest the β′ phase is not stable. (e) The structure of the β′-like transition state in *Path 2* (i.e., state III of Figure 4d in the main text). (f) The forces on atoms in the β′-like phase.

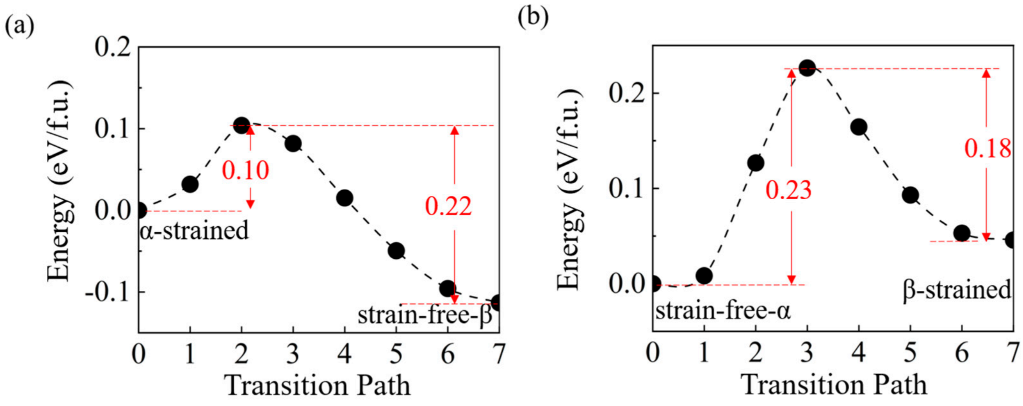

Figure S7. The energy landscapes of the phase transitions via *Path 1* under the two strained conditions: (a) from α-strained α-MnTe to strain-free β-MnTe (i.e., α-strained condition) and (b) from strain-free α-MnTe to β-strained β-MnTe (i.e., β-strained condition).

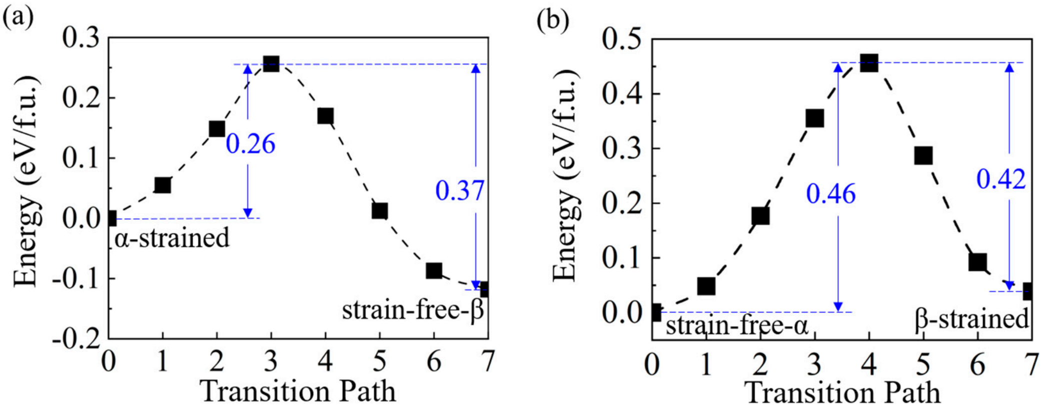

Figure S8. The energy landscapes of the phase transitions via *Path 2* under the two strained conditions: (a) from α-strained α-MnTe to strain-free β-MnTe (i.e., α-strained condition) and (b) from strain-free α-MnTe to β-strained β-MnTe (i.e., β-strained condition).

References

1. Kittel, C.; McEuen, P. *Introduction to solid state physics*; John Wiley & Sons: 2018.
2. Mori, S.; Hatayama, S.; Shuang, Y.; Ando, D.; Sutou, Y. Reversible displacive transformation in MnTe polymorphic semiconductor. *Nat. Commun.* **2020**, *11*, 85.
3. Mori, S.; Ando, D.; Sutou, Y. Sequential two-stage displacive transformation from β to α via β′ phase in polymorphic MnTe film. *Mater. Des.* **2020**, *196*, 109141.
