# Supplementary material for: Atomic Pathways of Crystal-to-Crystal Transitions and Electronic Origins of Resistive Switching in MnTe for Ultralow-Power Memory
Source: Nanomaterials (Basel). 2025 Jan 31;15(3):231. doi: 10.3390/nano15030231 (PMC11820569; doi:10.3390/nano15030231)
Supplement: Supplementary file 1 [file nanomaterials-15-00231-s001.zip › WR_Supplementary.docx]

Supplementary Material

**Atomic Pathways of Crystal-to-Crystal Transitions and Electronic Origins of Resistive Switching in MnTe for Ultralow-Power Memory**

Rui Wu^1^, Nian-Ke Chen^1,*^, Ming-Yu Ma^1^, Bai-Qian Wang^1^, Yu-Ting Huang^1^, Bin Zhang^2^, and Xian-Bin Li^1,*^

*^1^State Key Laboratory of Integrated Optoelectronics, College of Electronic Science and Engineering, Jilin University, Changchun 130012, China*

*^2^Analytical and Testing Center, Chongqing University, Chongqing 401331, China*

**Author to whom any correspondence should be addressed.*

*Email: [chennianke@jlu.edu.cn](mailto:chennianke@jlu.edu.cn) or [lixianbin@jlu.edu.cn](mailto:lixianbin@jlu.edu.cn)

**Table S1.** The magnetic moments at the Mn sites for α-MnTe and β-MnTe. The unit is $\mu_{B}$.

| α-MnTe | | β-MnTe | |
| --- | --- | --- | --- |
| Mn1 | 4.438 | Mn1 | 4.505 |
| Mn2 | -4.421 | Mn2 | -4.505 |


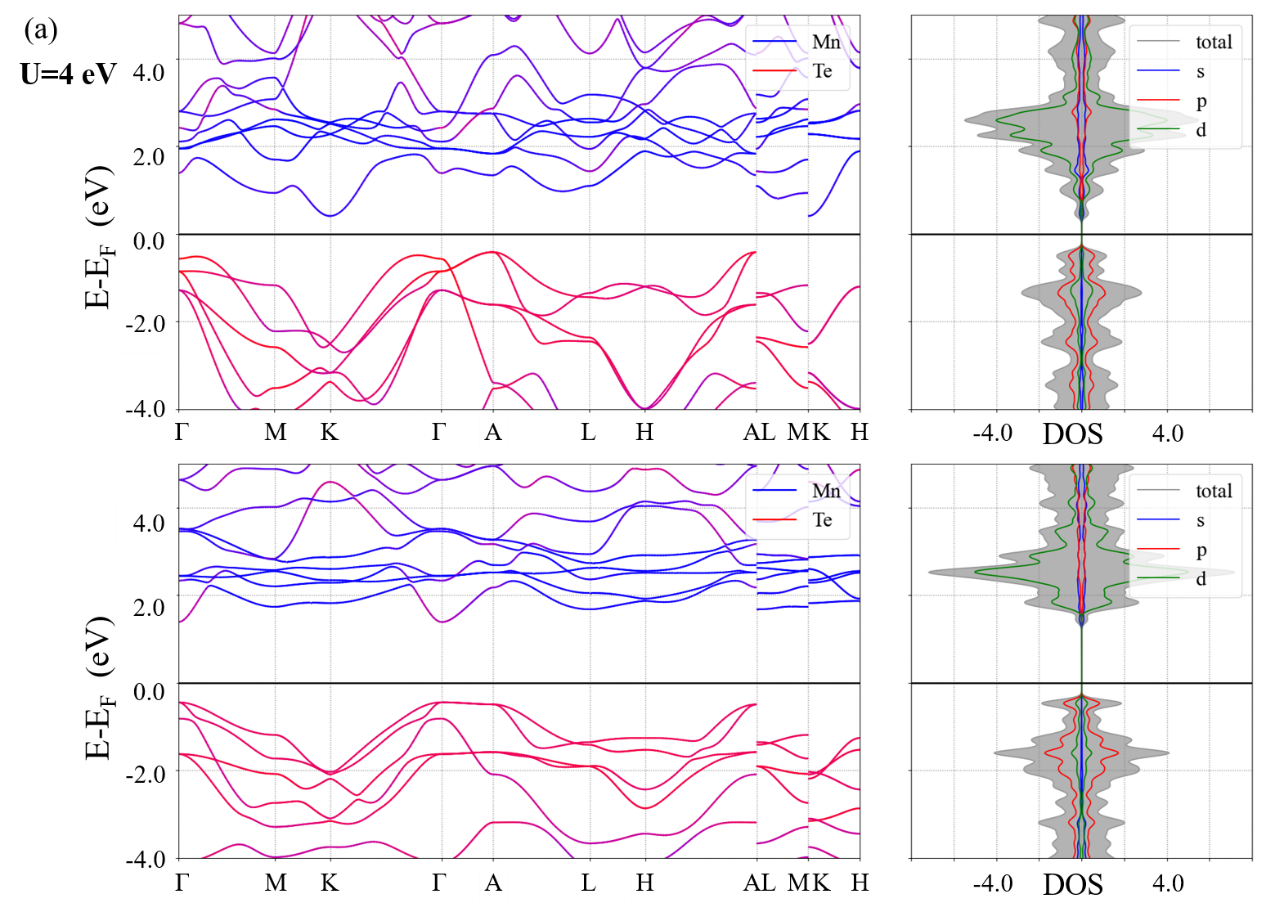

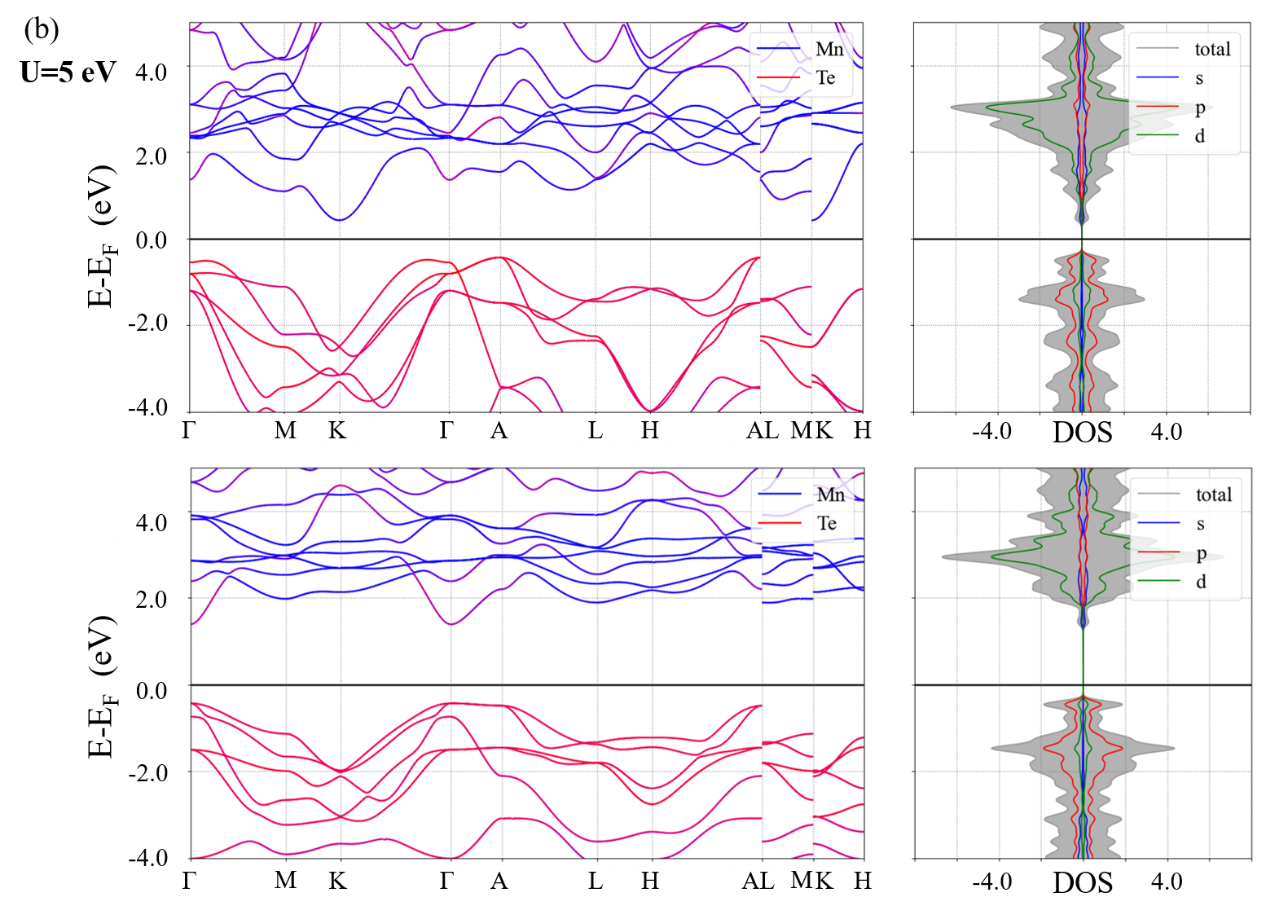


**Figure S1.** (a)The band structures and density of states of α-MnTe and β-MnTe calculated using GGA+U (U = 4 eV) method. (b)The band structures and density of states of α-MnTe and β-MnTe calculated using GGA+U (U = 5 eV) method.

**Table S2.** The bandgap and total energy of MnTe calculated using different values of U.

|  | α-bandgap (eV) | β-bandgap (eV) | Eα (eV) | Eβ (eV) |
| --- | --- | --- | --- | --- |
| U = 3 eV | 0.76 | 1.84 | -22.72 | -22.76 |
| U = 4 eV | 0.82 | 1.83 | -22.18 | -22.26 |
| U = 5 eV | 0.85 | 1.82 | -21.71 | -21.80 |


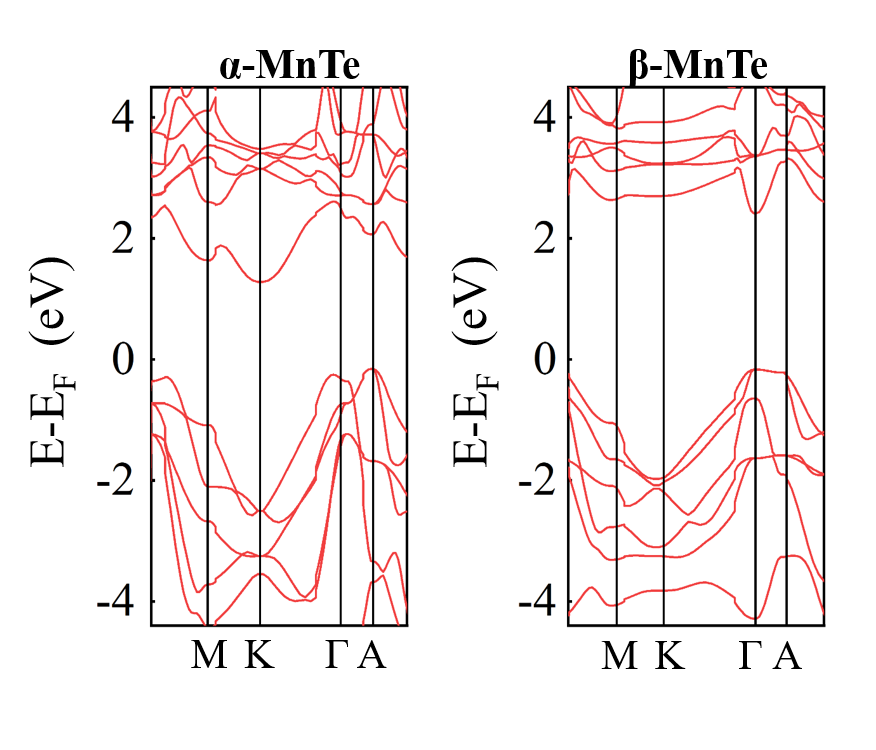


**Figure S2.** The band structures of α-MnTe and β-MnTe calculated using the HSE06 method.


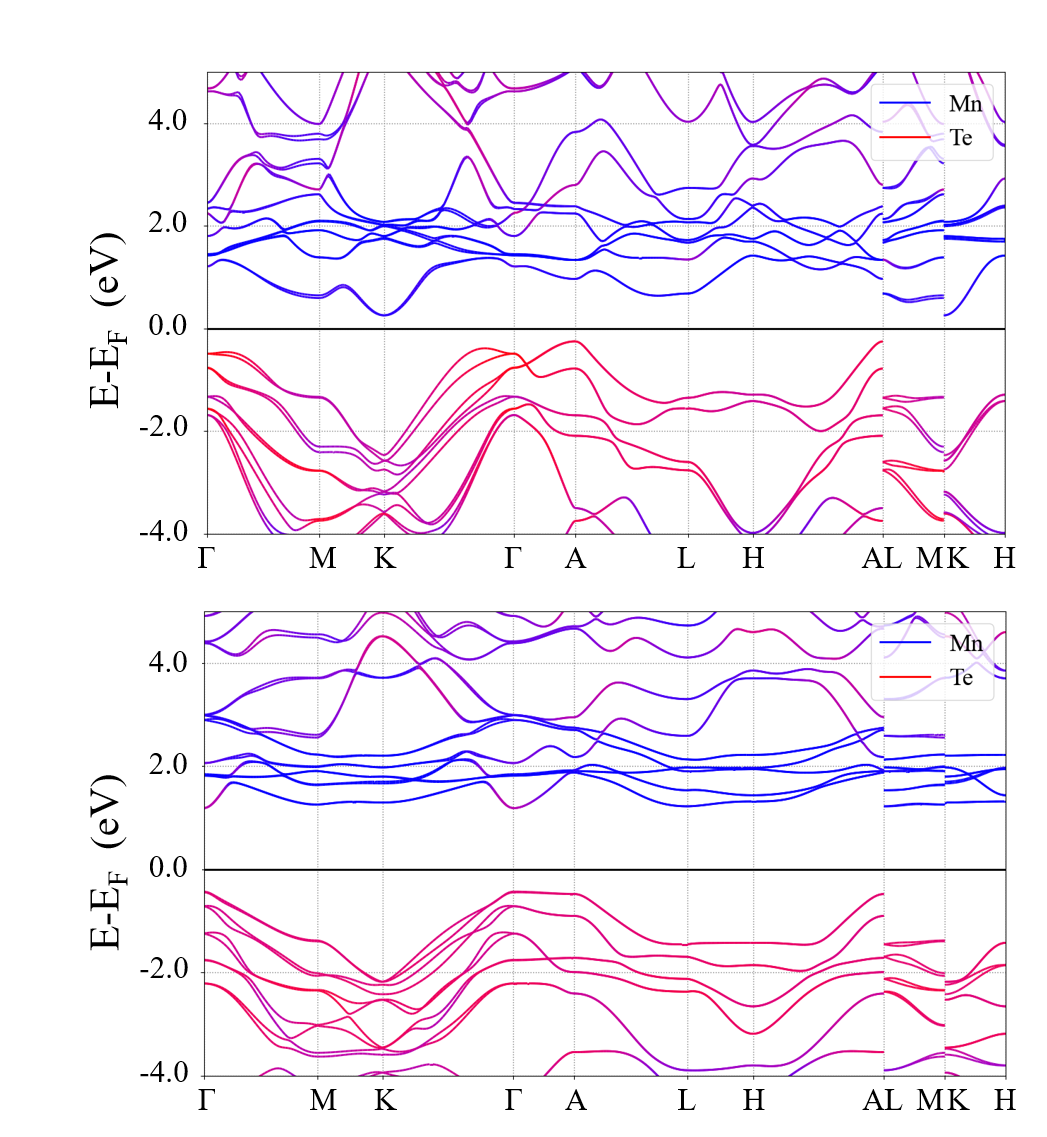


**Figure S3.** Band structures of α-MnTe and β-MnTe calculated with the spin-orbit coupling (SOC) effect.

The effective mass tensor can be expressed as

$\frac{d^{2}E}{dk^{2}}=\left( \begin{matrix} \frac{d^{2}E}{dk_{x}^{2}} & \frac{d^{2}E}{dk_{x}dk_{y}} & \frac{d^{2}E}{dk_{x}dk_{z}} \\ \frac{d^{2}E}{dk_{x}dk_{y}} & \frac{d^{2}E}{dk_{y}^{2}} & \frac{d^{2}E}{dk_{y}dk_{z}} \\ \frac{d^{2}E}{dk_{x}dk_{z}} & \frac{d^{2}E}{dk_{y}dk_{z}} & \frac{d^{2}E}{dk_{z}^{2}} \end{matrix} \right)$,

where x, y, z are the directions in the reciprocal Cartesian space. The angle between the *k_x_* direction and the *k_y_* direction is 60 degrees for a hexagonal lattice. Eigenvalues of the above matrix are inverses of the effective masses, eigenvectors are the directions of the principal effective mass components ($m_{1}^{*}$, $m_{2}^{*}$, $m_{3}^{*}$). The eigenvectors of the principal effective masses (keeping one significant figure after the decimal point) are presented in Table S3.

**Table S3.** The corresponding eigenvectors of the principal effective masses.

|  | α-CBM | α-VBM | β-CBM | β-VBM |
| --- | --- | --- | --- | --- |
| $m_{1}^{*}$ | 0.352 | -0.326 | 0.209 | -0.915 |
| Eigenvectors for $m_{1}^{*}$ | [-0.9, 0.4, 0.0] | [0.0, 0.8, 0.6] | [-0.6, 0.4, -0.7] | [0.1, 1.0, 0.1] |
| $m_{2}^{*}$ | 0.352 | -0.459 | 0.058 | -0.918 |
| Eigenvectors for $m_{2}^{*}$ | [0.4, 0.9, 0.0] | [1.0, 0.0, 0.0] | [0.4, 0.9, 0.2] | [1.0, -0.1, 0.0] |
| $m_{3}^{*}$ | 0.259 | -3.120 | 0.042 | -7.550 |
| Eigenvectors for $m_{3}^{*}$ | [0.0, 0.0, 1.0] | [0.0, -0.6, 0.8] | [-0.7, 0.2, 0.7] | [0.0, 0.1, 1.0] |

**Note S1. Calculation details of formation energy and concentration of V_Mn_**

For both α-MnTe and β-MnTe, the supercells of 4×4×3 are used to include the defects of Mn vacancies. The neutral formation energy for a defect $d$ is defined as

$$\Delta H_{f} \left( d \right)=E\left( d \right)- E\left( host \right)+\sum_{i} n_{i}{(E}_{i}+\mu_{i})$$

where *E*$\left( d \right)$ is the total energy of the supercell containing a defect $d$, and $E(host)$ is the total energy of the perfect host supercell. $n_{i}$ is the number of atoms of element $i$ being exchanged during the defect’s formation between the host supercell and the atom reservoir with the energy $E_{i}+\mu_{i}$, where $E_{i}$ is the energy per atom in the stable phase of element $i$, and $\mu_{i}$ is the chemical potential with respect to $E_{i}$.

The chemical potentials of Mn ($\mu_{Mn}$) and Te ($\mu_{Te}$) atoms in MnTe are correlated by the enthalpy of formation of MnTe compounds $\Delta H\left( MnTe \right)$ with respect to the elementary substances, which is defined as

$\Delta H \left( MnTe \right)$ = $E\left( MnTe \right)- [E\left( Mn \right)+E\left( Te \right)]$,

where $E\left( MnTe \right)$ is the energy per formula unit of MnTe cell, $E\left( Mn \right)$ and $E\left( Te \right)$ are the energy per atom of Mn and Te elementary substances, respectively. The calculated $E\left( Mn \right)$ and $E\left( Te \right)$ are -5.33 eV/atom and -3.14 eV/atom, respectively. The calculated enthalpy of formation of α-MnTe and β-MnTe are -2.89 eV/f.u. and -2.91 eV/f.u., respectively.

Then, the chemical potentials of Mn and Te should satisfy the relationships of

$\Delta H \left( MnTe \right)=\mu_{Mn}+\mu_{Te}$,

$\mu_{Mn}\leq0$,

$\mu_{Te}\leq0$.

The upper limits of the chemical potentials are $\mu_{Te}=0$ or $\mu_{Mn}=0$, which correspond to the Mn-poor (Te-rich) or Mn-rich (Te-poor) conditions, respectively. Then we calculated the chemical potential-dependent formation energy of a V_Mn_ defect ($n_{i}$=1).

For α-MnTe, the formation energy of V_Mn_, $\Delta H_{f} \left( V_{Mn}\_\alpha\right)$ is calculated by

$\Delta H_{f} \left( V_{Mn}\_\alpha\right)=E\left( V_{Mn}\_\alpha\right)- E\left( host\_\alpha\right)+{(E}_{Mn}+\mu_{Mn}$),

$\Delta H \left( \alpha MnTe \right){\leq\mu}_{Mn}\leq0$.

Then we get

$\Delta H_{f} \left( V_{Mn}\_\alpha\right)=3.25+\mu_{Mn}$,

-2.89$\leq\mu_{Mn}\leq$0.

For β-MnTe, the formation energy of V_Mn_, $\Delta H_{f} \left( V_{Mn}\_\beta\right)$ is calculated by

$\Delta H_{f} \left( V_{Mn}\_\beta\right)=E\left( V_{Mn}\_\beta\right)- E\left( host\_\beta\right)+{(E}_{Mn}+\mu_{Mn}$),

$\Delta H \left( \beta MnTe \right){\leq\mu}_{Mn}\leq0$.

Then we get

$\Delta H_{f} \left( V_{Mn}\_\beta\right)=4.49+\mu_{Mn}$,

-2.91$\leq\mu_{Mn}\leq$0.

The results of the chemical potential-dependent formation energy of V_Mn_ are shown in Figure S4. The formation energies of V_Mn_ in α-MnTe are always larger than those in β-MnTe under the same conditions.

The number of vacancies (*n*) at equilibrium states can be estimated by the following equation ^s^[1]:

$$n=N\cdot exp(-\frac{E_{f}}{k_{B}T})$$

where N is the number of lattice sites of Mn,$E_{f}$is the formation energy of $V_{Mn}$, $k_{B}$ is the Boltzmann constant, T is the temperature. The formation energy of $V_{Mn}$ in β-MnTe ($E_{f\_\beta}$) is 1.24 eV lager than that ($E_{f\_\alpha}$) in α-MnTe (Figure S4). Then the ratio of the $V_{Mn}$ concentrations in β-MnTe and α-MnTe can be expressed as

$$\frac{n_{\beta}}{n_{\alpha}}=\frac{N\cdot exp(-\frac{E_{f\_\beta}}{k_{B}T})}{N\cdot exp(-\frac{E_{f\_\alpha}}{k_{B}T})}=\exp\left( -\frac{E_{f_{\beta}}-E_{f_{\alpha}}}{k_{B}T} \right)$$

Then the calculated results are shown in Figure S5. At equilibrium states, the $V_{Mn}$ concentration in β-MnTe is much smaller than that in α-MnTe.


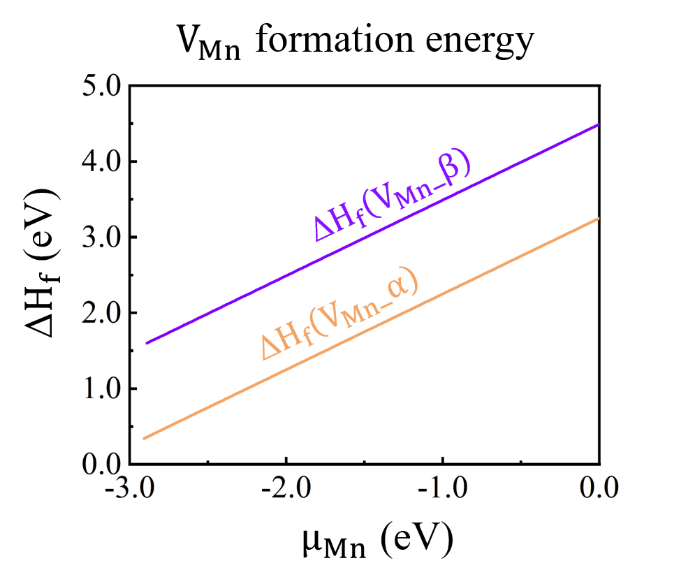


**Figure S4.** Neutral formation energy of Mn vacancy in α-MnTe and β-MnTe as a function of chemical potential $\mu_{Mn}$.


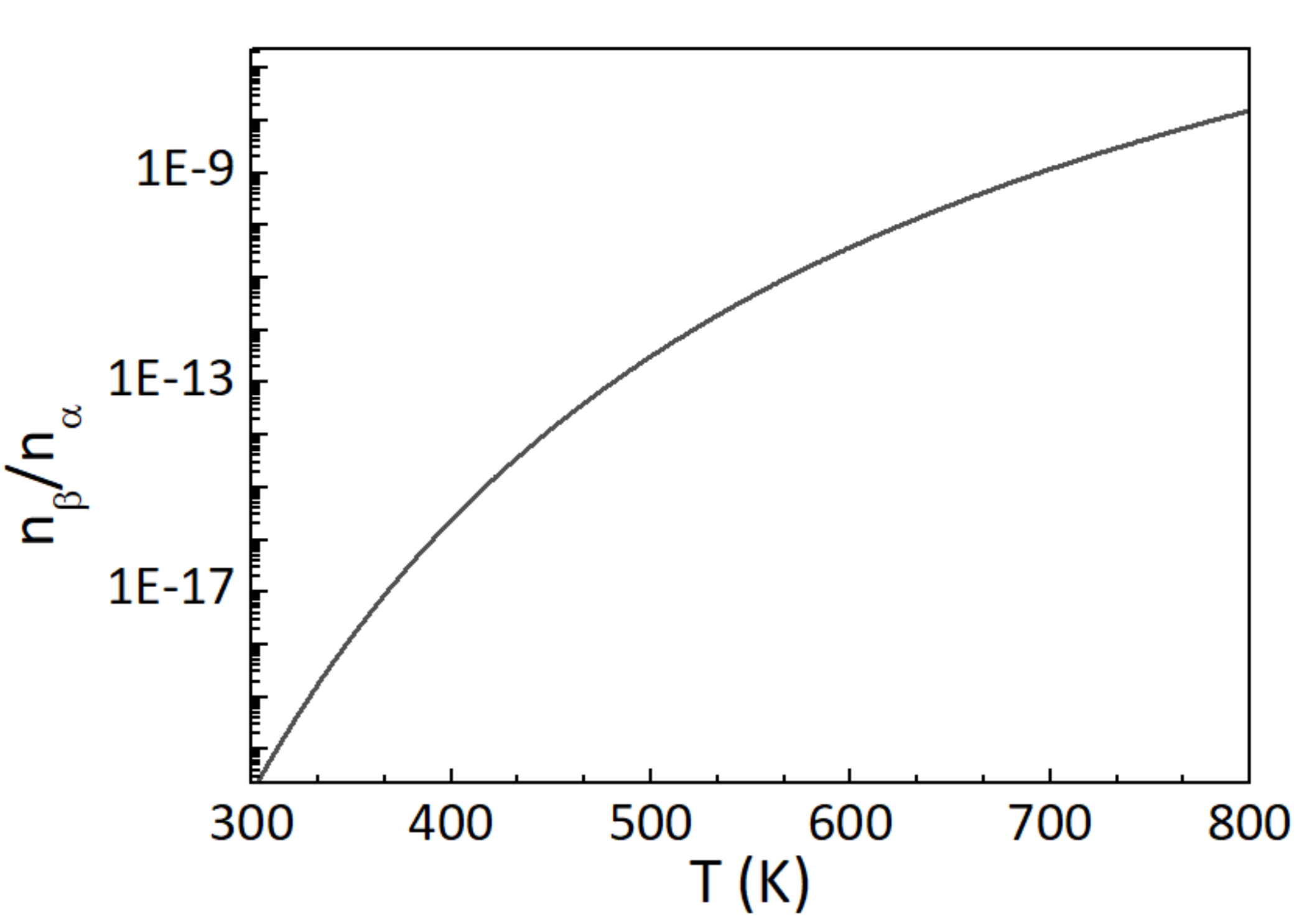


**Figure S5.** The ratio of the $V_{\mathrm{Mn}}$ concentrations in β-MnTe to that in α-MnTe as a function of temperature at their equilibrium states.


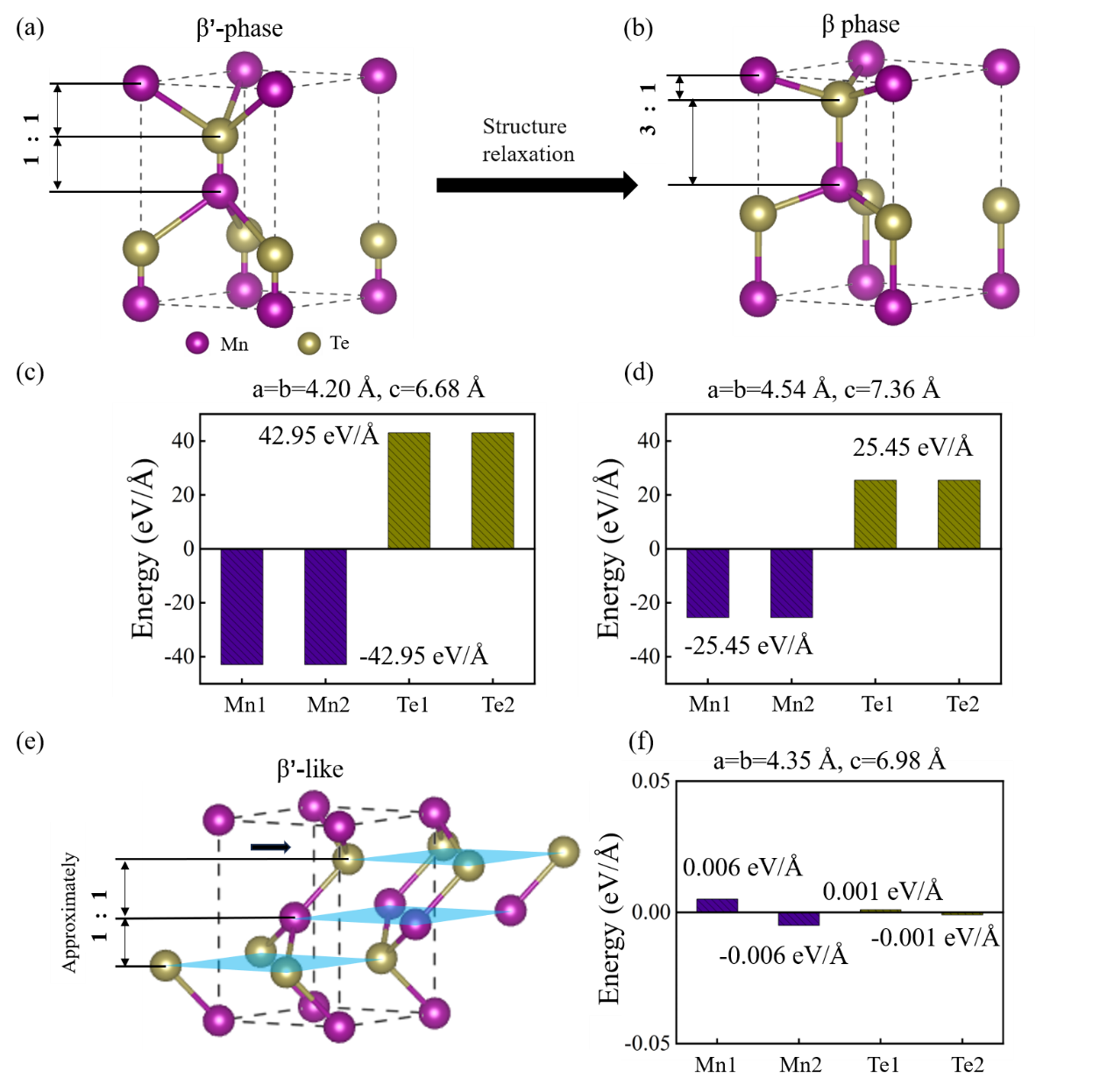


**Figure S6.** (a) The structure of the β*'* phase as depicted in the previous reports.^s^[2,3] The β*'* phase is defined as that the vertical distance between Mn and Te atoms along the c-axis is in the ratio of 1:1. However, after structural relaxation, the ratio becomes 3:1, which corresponds to (b) the standard β phase. The forces on Mn and Te atoms in β*'* phases with different lattice constants: (c) the c-axis lattice constant of β*'* phase adopts that of α phase, (d) the c-axis lattice constant of β*'* phase adopts that of β phase. The extremely large atomic forces suggest the β*'* phase is not stable. (e) The structure of the β*'*-like transition state in *Path 2* (i.e., state III of Figure 4d in the main text). (f) The forces on atoms in the β*'*-like phase.


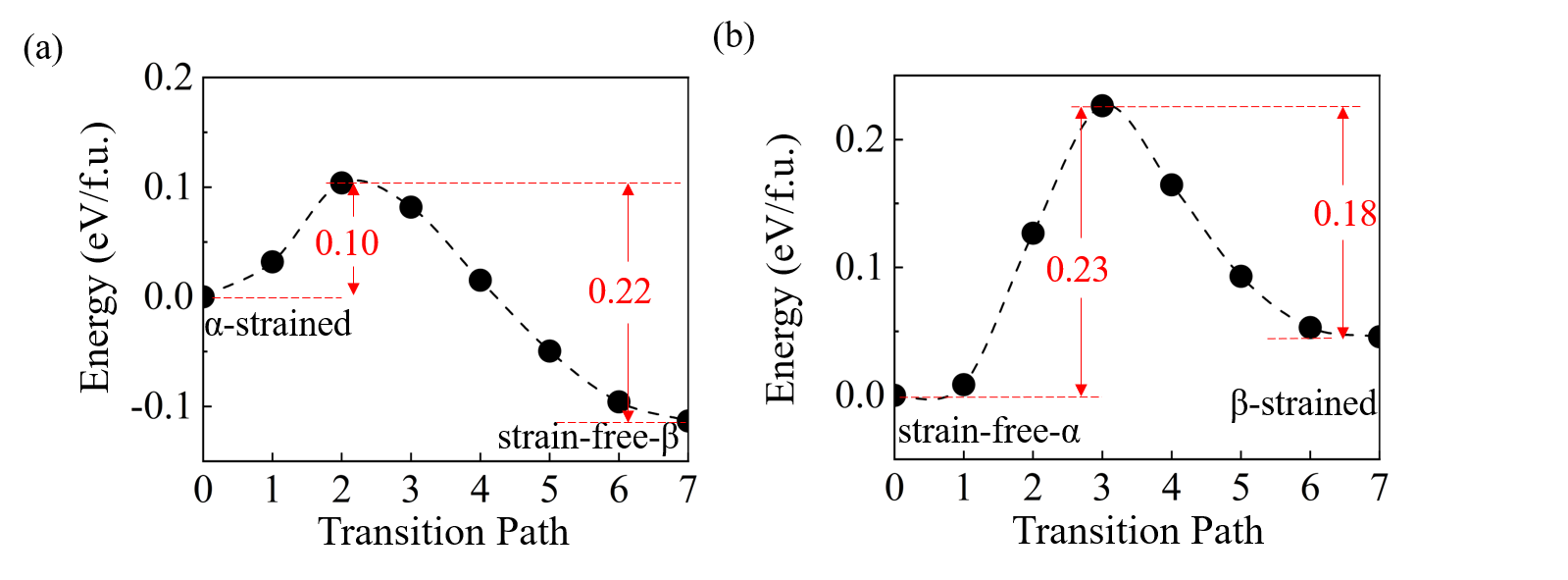


**Figure S7.** The energy landscapes of the phase transitions via *Path 1* under the two strained conditions: (a) from α-strained α-MnTe to strain-free β-MnTe (i.e., α-strained condition) and (b) from strain-free α-MnTe to β-strained β-MnTe (i.e., β-strained condition).


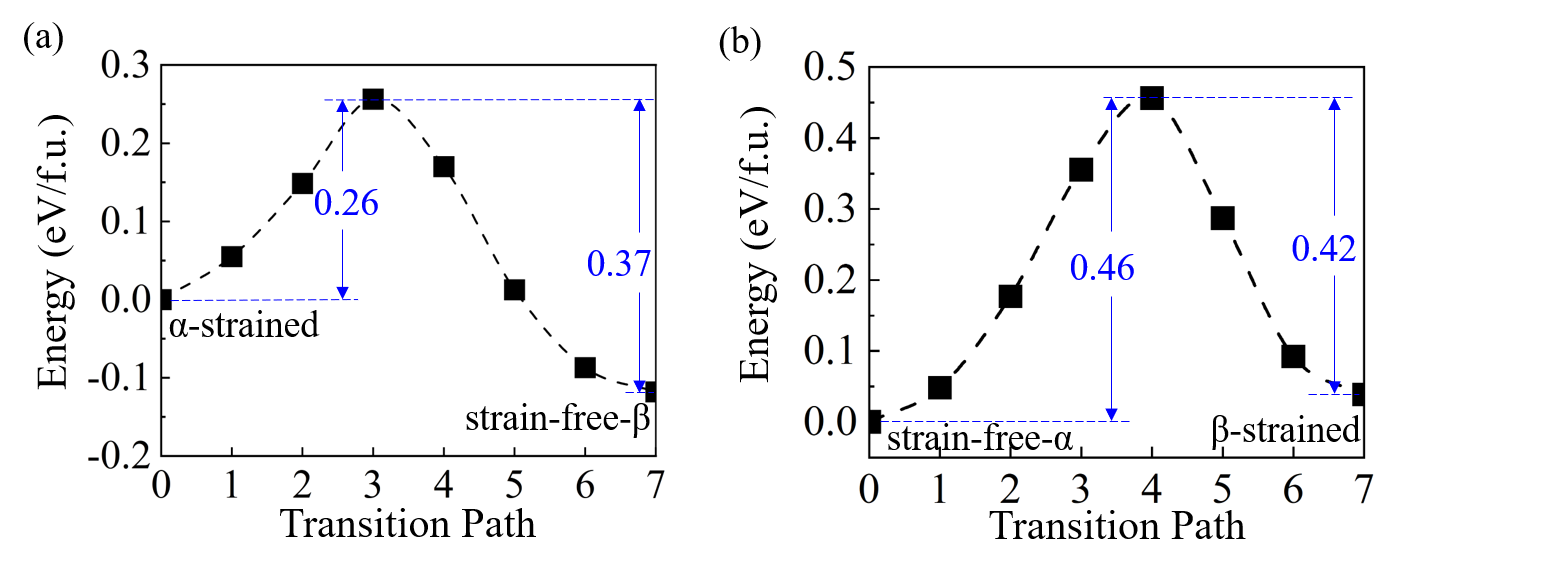


**Figure S8.** The energy landscapes of the phase transitions via *Path 2* under the two strained conditions: (a) from α-strained α-MnTe to strain-free β-MnTe (i.e., α-strained condition) and (b) from strain-free α-MnTe to β-strained β-MnTe (i.e., β-strained condition).

**References**

1. Kittel, C.; McEuen, P. *Introduction to solid state physics*; John Wiley & Sons: 2018.

2. Mori, S.; Hatayama, S.; Shuang, Y.; Ando, D.; Sutou, Y. Reversible displacive transformation in MnTe polymorphic semiconductor. *Nat. Commun.* **2020**, *11*, 85.

3. Mori, S.; Ando, D.; Sutou, Y. Sequential two-stage displacive transformation from β to α via β′ phase in polymorphic MnTe film. *Mater. Des.* **2020**, *196*, 109141.
